# Fragile X Syndrome as an interneuronopathy: a lesson for future studies and treatments

**DOI:** 10.3389/fnins.2023.1171895

**Published:** 2023-04-28

**Authors:** Alessandra Tempio, Asma Boulksibat, Barbara Bardoni, Sébastien Delhaye

**Affiliations:** ^1^Université Côte d’Azur, CNRS, Institut de Pharmacologie Moléculaire et Cellulaire, Valbonne, France; ^2^Inserm, Université Côte d’Azur, CNRS, Institut de Pharmacologie Moléculaire et Cellulaire, Valbonne, France

**Keywords:** interneurons, Fragile X Syndrome, interneuronopathy, FMRP, ASD, excitation/inhibition balance

## Abstract

Fragile X Syndrome (FXS) is the most common form of inherited intellectual disability (ID) and a primary genetic cause of autism spectrum disorder (ASD). FXS arises from the silencing of the *FMR1* gene causing the lack of translation of its encoded protein, the Fragile X Messenger RibonucleoProtein (FMRP), an RNA-binding protein involved in translational control and in RNA transport along dendrites. Although a large effort during the last 20  years has been made to investigate the cellular roles of FMRP, no effective and specific therapeutic intervention is available to treat FXS. Many studies revealed a role for FMRP in shaping sensory circuits during developmental critical periods to affect proper neurodevelopment. Dendritic spine stability, branching and density abnormalities are part of the developmental delay observed in various FXS brain areas. In particular, cortical neuronal networks in FXS are hyper-responsive and hyperexcitable, making these circuits highly synchronous. Overall, these data suggest that the excitatory/inhibitory (E/I) balance in FXS neuronal circuitry is altered. However, not much is known about how interneuron populations contribute to the unbalanced E/I ratio in FXS even if their abnormal functioning has an impact on the behavioral deficits of patients and animal models affected by neurodevelopmental disorders. We revise here the key literature concerning the role of interneurons in FXS not only with the purpose to better understand the pathophysiology of this disorder, but also to explore new possible therapeutic applications to treat FXS and other forms of ASD or ID. Indeed, for instance, the re-introduction of functional interneurons in the diseased brains has been proposed as a promising therapeutic approach for neurological and psychiatric disorders.

## Introduction

Fragile X Syndrome (FXS) is the most prevalent genetic form of intellectual disability, following an X-linked inheritance, associated with deficits in cognition, language, Autism Spectrum Disorder (ASD), anxiety, epilepsy and Attention Deficit Hyperactivity Disorder (ADHD) (Hagerman et al., 2017). In FXS, the *FMR1* gene is silenced and, consequently, its product, the Fragile X Messenger Ribonucleoprotein Protein (FMRP), is entirely absent. FMRP is an RNA-binding protein involved in different steps of mRNA metabolism, such as translational control both in soma and at the synaptic level, RNA transport along dendrites and from nucleus to cytoplasm (Maurin et al., 2014; Richter and Zhao, 2021; Kieffer et al., 2022). FMRP regulates the shaping of sensory circuits during the critical period, which is a time during early postnatal life when the development and maturation of functional properties of the brain is strongly dependent on experience or environmental influences. Indeed, early sensory activity is pivotal for the maturation of visual (Burbridge et al., 2014) and somatosensory networks (Tuncdemir et al., 2016). FMRP loss causes alterations in maturation and pruning of dendritic spines and dysregulates the expression of a large number of synaptic proteins, which are essential for the correct function of cerebral circuits (Richter and Zhao, 2021). The information flow between brain regions occurs due to a fine balance between excitatory and inhibitory neurons that control the output signal. Excitatory (E) and Inhibitory (I) synapses have different architectures. Pyramidal cells comprise the majority of the neuronal population and are primarily responsible for long-range glutamatergic transmission in the mammalian forebrain. GABAergic interneurons (INs) are the major inhibitory neurons in the central nervous system (Zhang et al., 2021), where they control and synchronize the synaptic activity of excitatory neurons. They represent 10–25% of the total number of cortical neurons and are classified based on their morphology, molecular markers, postsynaptic targets, origin area, electrophysiological properties and functions, according to the Petilla terminology (Ascoli et al., 2008). Cognition, behavior, and sensory information processing depends on this efficient balance. The control of neuronal excitability and ability of synapses to strengthen or weaken in response to an enhancement or decrease in their activity provide an efficient mechanism to tune up the E/I responses (Sears and Hewett, 2021). Synapses are extremely plastic structures, modifying their activity based on changes in neuronal activity or sensory experiences. Nevertheless, it is mandatory that these changes are synchronized with other synapses to maintain E/I inputs. Due to the fine regulation of the ratio between E/I synapses, its disruptions induce a broad range of neurological and psychiatric disorders, such as FXS. This pathology can be classified as an interneuronopathy, where an alteration in inhibitory activity occurs rendering some neuronal circuits hyper-responsive and hyper-excitable (Sohal and Rubenstein, 2019).

### The GABAergic inhibitory system is impaired in FXS

**Figure 1:**
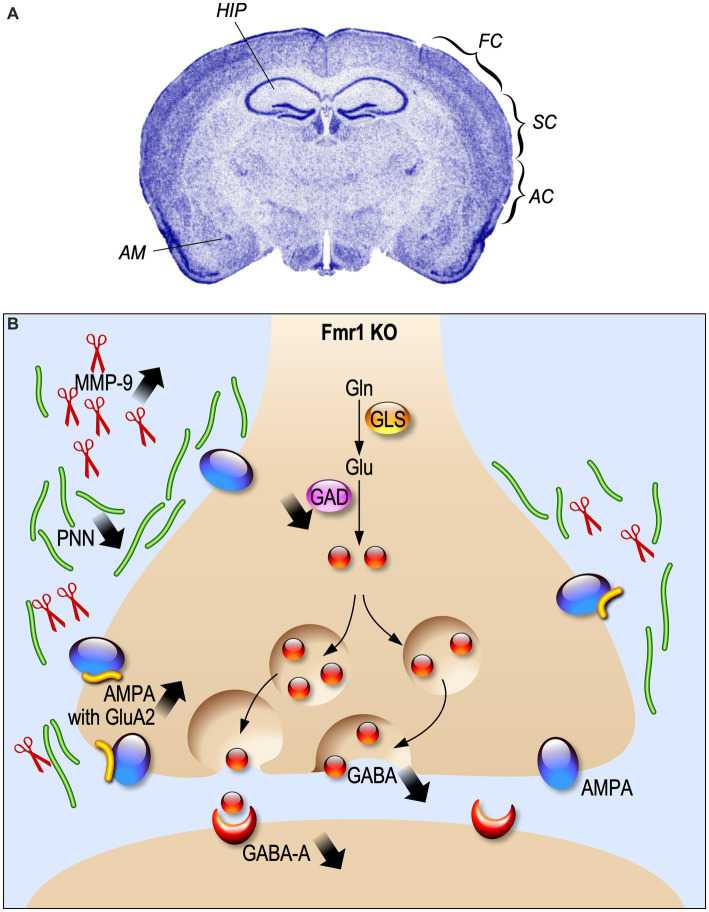
Electrophysiological alterations in Fragile X Syndrome. **(A)** Fmr1 KO brain regions where the inhibitory system is impaired are indicated. HIP, hippocampus; FC, frontal cortex; SC, somatosensory cortex; AC, auditory cortex; AM, amygdala. **(B)** Schematic representation of synaptic alteration in GABAergic synapses.

Most of the altered excitatory mechanisms in FXS are described in the framework of the mGluR theory, according to which the absence of FMRP exaggerates mGluR-dependent protein synthesis, leading to altered synaptic plasticity (Bear et al., 2004). However, FMRP is also expressed in GABAergic neurons at post-natal day 21 (PND 21) (Olmos-Serrano et al., 2010) and regulates the expression of different components of GABAergic transmission (Paluszkiewicz et al., 2011a). Indeed, GABA_A_ receptor δ subunits in neocortex are downregulated in adult *Fmr1* KO mice at age of 8–12 weeks (d'Hulst et al., 2006; Figure 1). In human patients, a reduction of the GABA_A_-mediated intracortical inhibition associated to an increase of intracortical circuit excitability was reported (Morin-Parent et al., 2019). Moreover, a decreased GABA concentration in the frontal cortex and thalamus of neonatal PND 5 *Fmr1* KO mice was found (Reyes et al., 2020). In line with the reduced excitability showed by INs, also the availability of GABA is decreased at PND 21 in the *Fmr1* KO amygdala, due to a decline in the number of inhibitory synapses and a reduced expression of GAD65/67, a rate-limiting enzyme for GABA synthesis (Olmos-Serrano et al., 2010; Figure 1). All these alterations lead to a hyper-activity of neuronal circuits that can explain the typical behavioral disturbances of FXS such as exaggerated fear, anxiety and hyperactivity (Figure 1).

In the somatosensory cortex of 1 year-old *Fmr1* KO mice, a reduction of parvalbumin (PV)-positive density, but not calbindin (CB) and calretinin (CR)-positive INs was described (Selby et al., 2007). In addition, PV interneurons present a bigger soma and an impaired distribution in the lamina. Interestingly, PV INs reduction mainly occurs in somatosensory cortical layers II/III/IV of 8-week-old *Fmr1* KO mice, but not in deeper layers V and VI where PV INs number is increased (Selby et al., 2007; Lee et al., 2019). The density of somatostatin (SOM)-positive INs in layer II/III does not change between WT and *Fmr1* KO mice at PND 19–31, as well as the proportion of layer II/III SOM/CR-positive INs (Paluszkiewicz et al., 2011b). Moreover, *Fmr1* KO fast-spiking (FS) INs display an immature dendritic morphology during the critical period at PND 5–6 (Nomura et al., 2017), while at PND 9–10 there are no differences compared to normal (Crair and Malenka, 1995). These interneuronal in impairments could result into an alteration in the physiological onset of critical period, cell migration, differentiation of neurons and refinement of neuronal connectivity (Hensch and Fagiolini, 2005; Luhmann et al., 2015; Begum and Sng, 2017).

Indeed, alterations in sensory experience processing, like in FXS, induce a disruptive development not only in synaptic plasticity of excitatory neurons, but also in cortical INs-afferent connectivity. This hypothesis is supported by the description of an alteration of cortical INs-afferent connectivity of the PVs and SOM cortical INs in PND 30 *Fmr1* KO mice (Pouchelon et al., 2021). The number of synapses and neurons is strongly regulated by experience influence during development. Layers I-IV of the auditory cortex present a developmental enhancement of PV cell density in both WT and *Fmr1* KO mice at PND 21, but *Fmr1* KO auditory cortex has less PV cell density than WT (Wen et al., 2018b). Like PV INs, perineuronal nets (PNNs), which are proteins in the extracellular matrix often associated with PV cells, show a developmental increase. However, *Fmr1* KO mice show a reduction of PNNs selectively at PND 21 in layer II-IV of the auditory cortex. This loss of PNNs around PV cells is associated with abnormal critical period plasticity and reduced excitability of PV cells (Figure 1).

The endopeptidase Matrix Metalloproteinase-9 (MMP-9) cleaves the extracellular matrix components of PNN and is over-expressed in *Fmr1* KO mice, leading to an altered PNN formation (Sidhu et al., 2014; Figure 1). The PNN pattern can be rescued by MMP-9 genetic deletion (Wen et al., 2018a) or by its pharmacological inhibition at PND 22 (Pirbhoy et al., 2020).

### Electrophysiological and Ca^2+^ alterations in FXS interneurons

**Figure 2:**
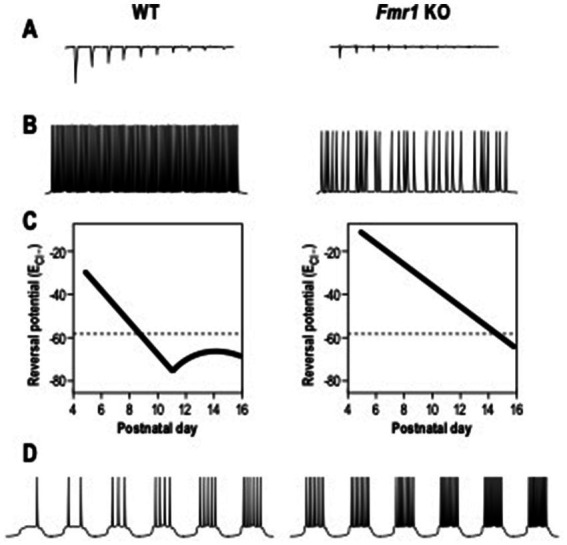
Electrophysiological alterations in Fragile X Syndrome. **(A)** The local excitation of interneurons, induced by excitatory neurons and measured as amplitude of excitatory post-synaptic currents, is reduced in *Fmr1* KO mice compared to WT mice. **(B)** The action potentials firing of interneurons induced by DHPG, agonist of group 1 metabotropic glutamate receptor (mGluR), is decreased in *Fmr1* KO mice. **(C)** The chloride reversal Potential (E_Cl_-) remains depolarized in excitatory neurons of *Fmr1* KO mice during neuronal development. **(D)** The firing action potential rates in excitatory neurons is increased in *Fmr1* KO mice than in WT.

The ElectroEncephaloGram (EEG) power represents the amount of neurons that fire synchronously in a certain frequency band (Willerman et al., 1991), while coherence is used to highlight if two or more brain regions have comparable oscillatory activity (Bowyer, 2016). In FXS patients, the resting-state EEG recordings showed an increased relative theta power (4–8 Hz), a reduced relative upper-alpha (10–12 Hz) and beta (12–30 Hz) power (Van der Molen and Van der Molen, 2013; van der Molen et al., 2014), and a heightened gamma frequency (30–80 Hz) band power (Wang et al., 2017). These alterations in EEG power are a readout of elevated excitatory cortical activity and a decrease of the inhibition process (Contractor et al., 2015; Chen et al., 2017; Ethridge et al., 2017; Donoghue et al., 2020; Guyon et al., 2021). Analogous EEGs are recorded in murine models of FXS. Indeed, adult *Fmr1* KO mice show an increased delta and gamma resting EEG power between 1.5 and 3 months of age (Lovelace et al., 2018; Wen et al., 2019). Consistent with these results, it was shown that *Fmr1* deletion in forebrain excitatory neurons affects neuronal oscillations, enhancing the resting EEG gamma power in the auditory cortex of mice at PND 60–70 (Lovelace et al., 2020). Higher theta oscillations and coherence in the slow gamma band were recorded in the hippocampus of *Fmr1* KO mice at 8 weeks of age (Arbab et al., 2018). In addition, adult *Fmr1* KO mice display a cortical reduction of sound-evoked gamma synchrony (Kulinich et al., 2020; Lovelace et al., 2020). Consistent with the human and mice EEG recordings, *Fmr1* KO rats display a reduction in alpha power and enhanced baseline of gamma power at 5 weeks of age (Kozono et al., 2020). This alteration in gamma band power is correlated to impairment in social and sensory processing and it is influenced by the abnormal activation and development of PV positive – fast-spiking (FS) interneurons. These types of neurons undergo in developmental maturation during the early postnatal days, displaying modifications in membrane capacitance (*C*_m_), input resistance (*R*_in_) and neuronal activity (Itami et al., 2007). PV – FS interneurons in the FXS somatosensory cortex show a delay in the development of their intrinsic membrane properties during the critical period (Nomura et al., 2017). Indeed, in *Fmr1* KO INs, *C*_m_ is significantly lower during the critical period, whereas *R*_in_ is higher compared to WT INs. Moreover, *Fmr1* KO FS interneurons show a delay in the maturation of their firing properties, displaying an adaptation on the spiking activity, while FS mature INs are characterized by a non-adaptive spiking pattern. During the neurodevelopmental period, the local excitation of PV-FS inhibitory neurons is also altered in *Fmr1* KO mice, showing a decrease in the neocortex (Gibson et al., 2008; Patel et al., 2013; Nomura et al., 2017). These neuronal and synaptic delays in neonatal *Fmr1* KO mice can be rescued by chronic administration of a TrkB receptor agonist between PND 1 and PND17 (Nomura et al., 2017; Figure 2A). Moreover, the GABA switch from depolarizing to hyperpolarizing currents is delayed in cortical neurons of *Fmr1* KO mice (He et al., 2014; Figure 2).

In addition, SOM- low threshold spiking (LTS) INs of *Fmr1* KO mice at PND 19–31 are less activated by the group 1 metabotropic glutamate receptor (mGluR), generating inhibitory synaptic events with a reduced frequency (Paluszkiewicz et al., 2011b; Figure 2). LTS INs also present unsynchronized activity with pyramidal neurons, leading to the conclusions that those disruptions in neuronal synchrony could be the effect of disrupted LTS IN activity.

Alterations in the primary visual cortex of *Fmr1* KO mice are also present at 6–8 weeks of age: PV INs display a reduced visually evoked activity with lower frequency of the calcium peak induced by a visual stimulus compared to WT cells (Goel et al., 2018). Hypersensitivity was also displayed in neurons of the auditory cortex of *Fmr1* KO mice (Rotschafer and Razak, 2013), showing an increased response to a stimulus than WT mice (Wen et al., 2018a). Consistent with the general hyper-activation of the auditory cortex, there is an expanded frequency tuning in *Fmr1* KO neurons, where sound responses become abnormally high between PND 14 and PND 21, suggesting that a higher number of neurons in the auditory cortex are activated by a stimulus at the same time. This enhancement in responses could be caused by an alteration of the interneuronal activity (Patel et al., 2013; Wen et al., 2018a). Indeed, a decreased number of PV INs and impairments in the perineuronal and extracellular matrix components were described in the auditory cortex (Wen et al., 2018a). The genetic reduction of MMP-9 restores the magnitude of auditory cortex response in *Fmr1* KO neurons at PND 19–23 to WT levels (Wen et al., 2018a). These findings demonstrate the pivotal role of extracellular matrix to control the development and the functions of GABAergic neurons.

Due to the connections between the cortex and the amygdala, a disrupted cortical spike synchronization could then affect amygdala neuronal activity, leading to hyper-responsivity (Olmos-Serrano et al., 2010; Prager et al., 2016). Indeed, a significant neuronal hyperexcitability in pyramidal neurons of the amygdala was shown in *Fmr1* KO mice at PND 20–30 (Olmos-Serrano et al., 2010; Figure 2). Those neurons had a higher action potential (AP) frequency in response to a series of depolarizing current steps and also showed a decreased threshold for AP generation compared to WT. The synaptic response can be rescued by bath application of the GABA agonist gaboxadol (THIP), indicating a deficit in inhibitory transmission. Moreover, adult *Fmr1* KO pyramidal neurons in the amygdala display a reduced amplitude and frequency of inhibitor post-synaptic currents (sIPSC) (Olmos-Serrano et al., 2010). In young mice, at PND 10, amygdala neurons in *Fmr1* KO show reduced sIPSC amplitude and frequency, increasing at PND 14 (Vislay et al., 2013). In contrast, at PND 16, sIPSC amplitude returned to WT level, but the frequency remained high. At PND 21, sIPSC amplitude and frequency returned to control levels. These results show alterations at specific developmental points of inhibitory neurotransmission in the *Fmr1* KO amygdala.

Conversely, the cerebellar absence of FMRP reduces the spontaneous firing rate of Purkinje neurons at PND 26–32, due to an increased GABA release from IN basket cells (Yang et al., 2020). This interneuronal hyperactivity is induced by an altered activity of Kv1.2, a potassium channel highly expressed in fast-spiking GABAergic neurons. The deletion of *Fmr1* induces higher Ca^2+^ transients because of a lower interneuronal expression of Kv1.2, leading to an over-inhibition of Purkinje neurons.

Inhibitory INs display a form of synaptic plasticity which is independent from the activation of the NMDA receptor for glutamate, due to Ca^2+^ influx through AMPA receptors (Kullmann and Lamsa, 2007). The Ca^2+^ permeability of AMPA relies on the absence of the GluA2 subunit in the structure of the receptors (Akgül and McBain, 2016). In *Fmr1* KO mice at 2–3 weeks of age, CA1 inhibitory INs present an increased expression of the GluA2 subunit in AMPA receptor, which induces a decreased inwardly rectification of AMPAR-mediated excitatory synaptic current and a higher rectification index at glutamatergic synapses onto inhibitory INs (Hwang et al., 2022; Figure 1).

Recently, an altered AMPA response of the *Fmr1* KO cell fraction, enriched in INs, was highlighted thanks to the use of Agonist-induced functional analysis and cell sorting (ai-FACS) (Castagnola et al., 2020). This innovative tool allows to sort living cells on the base of their response to Ca^2+^ concentration changes in real time, using a fluorescent indicator after the application of a pharmacological agent. These analyses resulted in the identification of altered interneuronal populations during the early post-natal development of *Fmr1* KO brain (PND 18). In particular a reduced number of *Fmr1* KO INs express *Meis2*, a transcription factor involved in ASD, at PND18 and this alteration was restored at PND19 (Castagnola et al., 2020). These results confirmed at the molecular level the presence of a transient altered interneuronal phenotype during early post-natal brain development in the absence of FMRP.

### Involvement of interneurons in the behavioral phenotype of FXS

*Fmr1* KO mice exhibit a cognitive deficit, autistic features and hyperactivity. Many studies investigated extensively the sensory phenotypes in both patients and animal models of FXS (Dölen et al., 2007; Knoth et al., 2014). In particular, these mice display increased sensory responses and impaired sound selectivity (Rotschafer and Razak, 2013). Altered expression of PV and PNN in amygdala, hippocampus and auditory cortex of *Fmr1* KO mice were showed to be linked to impaired tone-associated memory formation in adult mice following fear conditioning (Reinhard et al., 2019). Indeed, lower levels of PNN in amygdala and auditory cortex could be the cause of impaired tone-associated fear memory in *Fmr1* KO mice as well as a reduced PNN density in hippocampal CA2. In addition, auditory cortex PV cell density is decreased after fear conditioning in both WT and *Fmr1* KO mice, while it is increased during learning in hippocampal CA3 only in WT mice, indicating a link between tone-associated memory and PV cells. Impaired visual discrimination in FXS mice at 6–8 weeks of age was also shown to be correlated to decreased activity of PV INs and to an orientation tuning deficit of pyramidal neurons (Goel et al., 2018). Goel et al. used an excitatory DREADD strategy, targeting PV cells in *Fmr1* KO mice that restored their visually evoked response and learning capacity in a visual discrimination task. More recently, the selective deletion of the *Fmr1* gene in PV- and SOM- expressing cells in mice induced an aberrant behavioral phenotype in adult mice at 6–8 weeks of age (Kalinowska et al., 2022). Mice with PV *Fmr1*-lacking INs showed anxiety-like behavior, altered social behavior and dysregulated *de novo* protein synthesis. Conversely, *Fmr1* loss in SOM-expressing neurons did not result in behavioral abnormalities and did not significantly impact *de novo* protein synthesis. This suggests that PV cells alteration contribute more in the *Fmr1* KO impaired behavior.

Remarkably, increased PV levels and enhanced PNN formation in the auditory cortex of *Fmr1* KO mice following MMP-9 inhibition is correlated with decreased anxiety and hyperactivity during adolescence (PND 27–28) (Pirbhoy et al., 2020). Consistent with these findings, MMP-9 deletion in *Fmr1*/*Mmp-9* double KO mice at the age of 2 months ameliorates anxiety, tested in an open field task, and social interaction (Sidhu et al., 2014). Consistent with these results, the reduced level of MMP-9 in *Mmp9* ^+/−^ /*Fmr1* KO mice rescue abnormal sensory gating tested with pre-pulse inhibition (PPI) of acoustic startle response (Kokash et al., 2019). Interestingly, in 3-month old *Fmr1* KO mice, the altered PPI can be rescued by GABA_A_ activation by the GABA_A_ receptor agonist THIP, supporting the aberrant GABAergic transmission theory in FXS (Olmos-Serrano et al., 2011). Another evidence of an altered inhibition of GABA signaling in FXS is represented by audiogenic seizures in *Fmr1* KO mice, which consist in an extreme manifestation of auditory hypersensitivity after loud sound stimuli (Chen and Toth, 2001). This behavioral phenotype can be reversed by intraperitoneal administration of GABA_A,_ agonists to *Fmr1* KO mice at PND 21–25 (Heulens et al., 2012). Moreover, *Fmr1* KO mice exposed to passive sound postnatally (PND 9–21) have a significantly increased number of PV cells (Kulinich et al., 2020), showing again the correlation between INs and auditory cortex development.

## Conclusion and therapeutic perspectives

Overall, the studies we summarized here strongly suggest that FXS is a form of interneuropathy. However, to advance the research in the field several aspects could be taken into account to design future studies:

- To date, most of the studies have characterized FXS INs in adult mice (Olmos-Serrano et al., 2010; Paluszkiewicz et al., 2011b; Arbab et al., 2018; Goel et al., 2018; Kokash et al., 2019; Lee et al., 2019; Reinhard et al., 2019; Lovelace et al., 2020; Yang et al., 2020; Pouchelon et al., 2021; Kalinowska et al., 2022), while only a few studies have taken in consideration interneuronal impairment during the critical window of postnatal development (Nomura et al., 2017; Castagnola et al., 2020; Reyes et al., 2020; Rais et al., 2022). It would be interesting to study and compare various ages in *Fmr1* KO mice, which are associated to an altered function of INs through the different steps of neurodevelopment.
- The different brain areas have been studied differently: more attention has been paid to cortex (Selby et al., 2007; Gibson et al., 2008; Paluszkiewicz et al., 2011b; Patel et al., 2013; He et al., 2014; Nomura et al., 2017; Goel et al., 2018; Wen et al., 2018a,b; Lee et al., 2019; Pirbhoy et al., 2020; Reyes et al., 2020; Pouchelon et al., 2021) compared to other brain regions, such as the hippocampus (Arbab et al., 2018; Reinhard et al., 2019; Hwang et al., 2022), leading to missing molecular and behavioral information to understand the physiopathology of FXS.
- Another aspect that should be better considered in the future is the interneuronopathy in both sexes. Recently, it was shown that an altered activation of PV INs in mice during the critical period, especially in the limbic structures of the brain, has an impact on anxio-depressive behavior in adulthood (Banerjee et al., 2022). Indeed, adult male and female animals in which PV-positive INs have been activated during the critical period were less anxious and showed a reduction in despair-like behavior in adulthood. However, this reduction was dependent on the task and on the sex, leading to the conclusion that also the female phenotype should be taken into consideration in the behavioral test. FXS is a X-linked disorder, for this reason female *Fmr1^−/−^* mice are poorly studied since not representative of patients affected by this syndrome, however behavioral differences have been described in *Fmr1* KO females compared to males (Nolan et al., 2017) as well as sex differences in molecular pathways have been highlighted (Jiang et al., 2021). These results suggest that the analysis this underrepresented population could help in the full understanding of brain function.
- Even if multiple pre-clinical studies have been carried out, the impact of various drugs was only episodically tested on interneuronal-associated phenotypes, as in the case of the modulation of TrkB or MMP-9 in infant *Fmr1* KO brain (Nomura et al., 2017; Pirbhoy et al., 2020).

The use of compounds directly linked to the GABAergic system (e.g., Baclofen, R-Baclofen and Ganaxolone that are GABA_B_ agonist) has been shown to rescue some of the molecular and behavioral phenotypes which characterize FXS in patients and in murine models (Heulens et al., 2012; Schaefer et al., 2015; Veenstra-VanderWeele et al., 2017; Jonak et al., 2022), suggesting that the rectification of the E/I imbalance through an enhancement of the GABAergic system could be a potential treatment for FXS. Although positive results were obtained in preclinical studies and in a Phase II clinical trial, these therapeutic approaches did not result into a broad treatment for FXS patients (Castagnola et al., 2017).

Due to the absence of significant results from the clinical studies, it remains a challenge to increase GABAergic system activity in those interneuropathies characterized by an excessive reduction in the GABA response. Among the drugs currently available we can mention metformin, an anti-hyperglycemic drug prescribed against diabetes mellitus type 2. The off-label use of metformin in FXS children improves language development and behavior (Biag et al., 2019). Furthermore, chronic treatment with metformin for 10 days in adult *Fmr1* KO mice rescues different behavioral deficits, such as social deficits and repetitive behavior and normalizes the over-expression of MMP-9 (Gantois et al., 2017). We hypothesize that metformin could have an effect also on IN development and maturation due to its effect on MMP-9 expression. In addition, cannabidiol has a positive allosteric modulation on GABA_A_ receptors (Bakas et al., 2017), enhancing GABAergic transmission, and improves the balance in inhibitory and excitatory transmission, restoring neuronal function and synaptic plasticity in patients with FXS (Palumbo et al., 2023).

Furthermore, a useful tool used to increase synaptic inhibition could be neuronal transplantation, which has the effect to improve the behavioral phenotype in several nervous system pathologies. In Alzheimer’s disease-related mouse models, transplanted embryonic IN progenitors restore normal cognitive functions (Tong et al., 2014). Moreover, the replacement of INs improves memory precision after traumatic brain injury, showing to be a powerful therapeutic strategy for correcting post-traumatic memory and seizure disorders (Zhu et al., 2019). In the same path, preclinical studies performed on an epilepsy animal model highlighted a reduction of seizures after transplantation of GABAergic INs or their progenitors (Cunningham et al., 2014; Hammad et al., 2015). In this context, it is interesting to underline that human induced pluripotent stem cell (iPSC)-derived cortical neurons were transplanted into the adult mouse cortex with human synaptic networks substantially restructured over 4 months, suggesting the potential usefulness of this technology (Real et al., 2018). Thus, the precise definition of affected INs subtypes during development in FXS, as well as in other forms of brain developmental disorders, could provide a new therapeutic approach for the most severe forms of developmental brain disorders. To reach this goal, single-cell sequencing and spatial omics technologies will be very useful in combination with functional analyses.

## Author contributions

AT: writing—original draft. AT, AB, BB, and SD: writing—review and editing. All authors contributed to the article and approved the submitted version.

## Funding

BB was supported by Agence Nationale de la Recherche ANR-20-CE16-0016 and ANR-15-IDEX-0001, Fondation Jérôme Lejeune (Project #2023) and Fondation de France (WB-2022-46003). AT was supported by FRAXA Research Foundation and SD was supported by Fondation Française pour la Recherche sur l’Épilepsie.

## Conflict of interest

The authors declare that the research was conducted in the absence of any commercial or financial relationships that could be construed as a potential conflict of interest.

## Publisher’s note

All claims expressed in this article are solely those of the authors and do not necessarily represent those of their affiliated organizations, or those of the publisher, the editors and the reviewers. Any product that may be evaluated in this article, or claim that may be made by its manufacturer, is not guaranteed or endorsed by the publisher.

## References

[ref1] Akgül G. McBain C. J. (2016). Diverse roles for ionotropic glutamate receptors on inhibitory interneurons in developing and adult brain. J. Physiol. 594, 5471–5490. doi: 10.1113/jp271764, PMID: 26918438 PMC5043048

[ref2] Arbab T. Battaglia F. P. Pennartz C. M. A. Bosman C. A. (2018). Abnormal hippocampal theta and gamma hypersynchrony produces network and spike timing disturbances in the Fmr1-KO mouse model of fragile X syndrome. Neurobiol. Dis. 114, 65–73. doi: 10.1016/j.nbd.2018.02.011, PMID: 29486296

[ref3] Ascoli G. A. Alonso-Nanclares L. Anderson S. A. Barrionuevo G. Benavides-Piccione R. Burkhalter A.. (2008). Petilla terminology: nomenclature of features of GABAergic interneurons of the cerebral cortex. Nat. Rev. Neurosci. 9, 557–568. doi: 10.1038/nrn2402, PMID: 18568015 PMC2868386

[ref4] Bakas T. Van Nieuwenhuijzen P. Devenish S. McGregor I. Arnold J. Chebib M. (2017). The direct actions of cannabidiol and 2-arachidonoyl glycerol at GABAA receptors. Pharmacol. Res. 119, 358–370. doi: 10.1016/j.phrs.2017.02.022 28249817

[ref5] Banerjee T. Pati S. Tiwari P. Vaidya V. A. (2022). Chronic hM3Dq-DREADD-mediated chemogenetic activation of parvalbumin-positive inhibitory interneurons in postnatal life alters anxiety and despair-like behavior in adulthood in a task- and sex-dependent manner. J. Biosci. 47:68. doi: 10.1007/s12038-022-00308-0 36476774

[ref6] Bear M. F. Huber K. M. Warren S. T. (2004). The mGluR theory of fragile X mental retardation. Trends Neurosci. 27, 370–377. doi: 10.1016/j.tins.2004.04.009, PMID: 15219735

[ref7] Begum M. R. Sng J. C. (2017). Molecular mechanisms of experience-dependent maturation in cortical GABA ergic inhibition. J. Neurochem. 142, 649–661. doi: 10.1111/jnc.14103, PMID: 28628196 PMC5599941

[ref8] Biag H. M. B. Potter L. A. Wilkins V. Afzal S. Rosvall A. Salcedo-Arellano M. J.. (2019). Metformin treatment in young children with fragile X syndrome. Mol. Genet. Genomic Med. 7:e956. doi: 10.1002/mgg3.956, PMID: 31520524 PMC6825840

[ref9] Bowyer S. M. (2016). Coherence a measure of the brain networks: past and present. Neuropsychiatr. Electrophysiol. 2, 1–12. doi: 10.1186/s40810-015-0015-7

[ref10] Burbridge T. J. Xu H.-P. Ackman J. B. Ge X. Zhang Y. Ye M.-J.. (2014). Visual circuit development requires patterned activity mediated by retinal acetylcholine receptors. Neuron 84, 1049–1064. doi: 10.1016/j.neuron.2014.10.051, PMID: 25466916 PMC4258148

[ref11] Castagnola S. Bardoni B. Maurin T. (2017). The search for an effective therapy to treat fragile X syndrome: dream or reality? Front. Synaptic Neurosci. 9:15. doi: 10.3389/fnsyn.2017.00015, PMID: 29163124 PMC5681520

[ref12] Castagnola S. Cazareth J. Lebrigand K. Jarjat M. Magnone V. Delhaye S.. (2020). Agonist-induced functional analysis and cell sorting associated with single-cell transcriptomics characterizes cell subtypes in normal and pathological brain. Genome Res. 30, 1633–1642. doi: 10.1101/gr.262717.120, PMID: 32973039 PMC7605246

[ref13] Chen L. Toth M. (2001). Fragile X mice develop sensory hyperreactivity to auditory stimuli. Neuroscience 103, 1043–1050. doi: 10.1016/S0306-4522(01)00036-7, PMID: 11301211

[ref14] Chen G. Zhang Y. Li X. Zhao X. Ye Q. Lin Y.. (2017). Distinct inhibitory circuits orchestrate cortical beta and gamma band oscillations. Neuron 96, 1403–1418.e6. doi: 10.1016/j.neuron.2017.11.033, PMID: 29268099 PMC5864125

[ref15] Contractor A. Klyachko V. A. Portera-Cailliau C. (2015). Altered neuronal and circuit excitability in fragile X syndrome. Neuron 87, 699–715. doi: 10.1016/j.neuron.2015.06.017 26291156 PMC4545495

[ref16] Crair M. C. Malenka R. C. (1995). A critical period for long-term potentiation at thalamocortical synapses. Nature 375, 325–328. doi: 10.1038/375325a0, PMID: 7753197

[ref17] Cunningham M. Cho J.-H. Leung A. Savvidis G. Ahn S. Moon M.. (2014). hPSC-derived maturing GABAergic interneurons ameliorate seizures and abnormal behavior in epileptic mice. Cell Stem Cell 15, 559–573. doi: 10.1016/j.stem.2014.10.006, PMID: 25517465 PMC4270101

[ref18] d'Hulst C. De Geest N. Reeve S. P. Van Dam D. De Deyn P. P. Hassan B. A.. (2006). Decreased expression of the GABAA receptor in fragile X syndrome. Brain Res. 1121, 238–245. doi: 10.1016/j.brainres.2006.08.115 17046729

[ref19] Dölen G. Osterweil E. Rao B. S. Smith G. B. Auerbach B. D. Chattarji S.. (2007). Correction of fragile X syndrome in mice. Neuron 56, 955–962. doi: 10.1016/j.neuron.2007.12.001, PMID: 18093519 PMC2199268

[ref20] Donoghue T. Haller M. Peterson E. J. Varma P. Sebastian P. Gao R.. (2020). Parameterizing neural power spectra into periodic and aperiodic components. Nat. Neurosci. 23, 1655–1665. doi: 10.1038/s41593-020-00744-x, PMID: 33230329 PMC8106550

[ref21] Ethridge L. E. White S. P. Mosconi M. W. Wang J. Pedapati E. V. Erickson C. A.. (2017). Neural synchronization deficits linked to cortical hyper-excitability and auditory hypersensitivity in fragile X syndrome. Mol. Autism. 8:22. doi: 10.1186/s13229-017-0140-1, PMID: 28596820 PMC5463459

[ref22] Gantois I. Khoutorsky A. Popic J. Aguilar-Valles A. Freemantle E. Cao R.. (2017). Metformin ameliorates core deficits in a mouse model of fragile X syndrome. Nat. Med. 23, 674–677. doi: 10.1038/nm.4335 28504725

[ref23] Gibson J. R. Bartley A. F. Hays S. A. Huber K. M. (2008). Imbalance of neocortical excitation and inhibition and altered UP states reflect network hyperexcitability in the mouse model of fragile X syndrome. J. Neurophysiol. 100, 2615–2626. doi: 10.1152/jn.90752.2008, PMID: 18784272 PMC2585391

[ref24] Goel A. Cantu D. A. Guilfoyle J. Chaudhari G. R. Newadkar A. Todisco B.. (2018). Impaired perceptual learning in a mouse model of fragile X syndrome is mediated by parvalbumin neuron dysfunction and is reversible. Nat. Neurosci. 21, 1404–1411. doi: 10.1038/s41593-018-0231-0, PMID: 30250263 PMC6161491

[ref25] Guyon N. Zacharias L. R. Fermino de Oliveira E. Kim H. Leite J. P. Lopes-Aguiar C.. (2021). Network asynchrony underlying increased broadband gamma power. J. Neurosci. 41, 2944–2963. doi: 10.1523/jneurosci.2250-20.2021, PMID: 33593859 PMC8018896

[ref26] Hagerman R. J. Berry-Kravis E. Hazlett H. C. Bailey D. B. Jr. Moine H. Kooy R. F.. (2017). Fragile X syndrome. Nat. Rev. Dis. Primers. 3:17065. doi: 10.1038/nrdp.2017.65 28960184

[ref27] Hammad M. Schmidt S. L. Zhang X. Bray R. Frohlich F. Ghashghaei H. T. (2015). Transplantation of GABAergic interneurons into the neonatal primary visual cortex reduces absence seizures in stargazer mice. Cereb. Cortex 25, 2970–2979. doi: 10.1093/cercor/bhu094, PMID: 24812085 PMC4537440

[ref28] He Q. Nomura T. Xu J. Contractor A. (2014). The developmental switch in GABA polarity is delayed in fragile X mice. J. Neurosci. 34, 446–450. doi: 10.1523/JNEUROSCI.4447-13.2014, PMID: 24403144 PMC6608154

[ref29] Hensch T. K. Fagiolini M. (2005). Excitatory–inhibitory balance and critical period plasticity in developing visual cortex. Prog. Brain Res. 147, 115–124. doi: 10.1016/S0079-6123(04)47009-5, PMID: 15581701

[ref30] Heulens I. D’Hulst C. Van Dam D. De Deyn P. P. Kooy R. F. (2012). Pharmacological treatment of fragile X syndrome with GABAergic drugs in a knockout mouse model. Behav. Brain Res. 229, 244–249. doi: 10.1016/j.bbr.2012.01.031, PMID: 22285772

[ref31] Hwang J. Y. Monday H. R. Yan J. Gompers A. Buxbaum A. R. Sawicka K. J.. (2022). CPEB3-dependent increase in GluA2 subunits impairs excitatory transmission onto inhibitory interneurons in a mouse model of fragile X. Cell Rep. 39:110853. doi: 10.1016/j.celrep.2022.110853, PMID: 35675768 PMC9671216

[ref32] Itami C. Kimura F. Nakamura S. (2007). Brain-derived neurotrophic factor regulates the maturation of layer 4 fast-spiking cells after the second postnatal week in the developing barrel cortex. J. Neurosci. 27, 2241–2252. doi: 10.1523/JNEUROSCI.3345-06.2007, PMID: 17329421 PMC6673466

[ref33] Jiang A. Wang L. Lu J. Y. Freeman A. Campbell C. Su P.. (2021). Sex differences in dopamine receptor signaling in fmr1 knockout mice: a pilot study. Brain Sci. 11:1398. doi: 10.3390/brainsci11111398, PMID: 34827397 PMC8615700

[ref34] Jonak C. R. Pedapati E. V. Schmitt L. M. Assad S. A. Sandhu M. S. DeStefano L.. (2022). Baclofen-associated neurophysiologic target engagement across species in fragile X syndrome. J. Neurodev. Disord. 14:52. doi: 10.1186/s11689-022-09455-9, PMID: 36167501 PMC9513876

[ref35] Kalinowska M. van der Lei M. B. Kitiashvili M. Mamcarz M. Oliveira M. M. Longo F.. (2022). Deletion of Fmr1 in parvalbumin-expressing neurons results in dysregulated translation and selective behavioral deficits associated with fragile X syndrome. Mol. Autism. 13:29. doi: 10.1186/s13229-022-00509-2, PMID: 35768828 PMC9245312

[ref36] Kieffer F. Hilal F. Gay A. S. Debayle D. Pronot M. Poupon G.. (2022). Combining affinity purification and mass spectrometry to define the network of the nuclear proteins interacting with the N-terminal region of FMRP. Front. Mol. Biosci. 9:954087. doi: 10.3389/fmolb.2022.954087, PMID: 36237573 PMC9553004

[ref37] Knoth I. S. Vannasing P. Major P. Michaud J. L. Lippé S. (2014). Alterations of visual and auditory evoked potentials in fragile X syndrome. Int. J. Dev. Neurosci. 36, 90–97. doi: 10.1016/j.ijdevneu.2014.05.003, PMID: 24875778

[ref38] Kokash J. Alderson E. M. Reinhard S. M. Crawford C. A. Binder D. K. Ethell I. M.. (2019). Genetic reduction of MMP-9 in the Fmr1 KO mouse partially rescues prepulse inhibition of acoustic startle response. Brain Res. 1719, 24–29. doi: 10.1016/j.brainres.2019.05.029, PMID: 31128097 PMC6640842

[ref39] Kozono N. Okamura A. Honda S. Matsumoto M. Mihara T. (2020). Gamma power abnormalities in a Fmr1-targeted transgenic rat model of fragile X syndrome. Sci. Rep. 10:18799. doi: 10.1038/s41598-020-75893-x, PMID: 33139785 PMC7608556

[ref40] Kulinich A. O. Reinhard S. M. Rais M. Lovelace J. W. Scott V. Binder D. K.. (2020). Beneficial effects of sound exposure on auditory cortex development in a mouse model of fragile X syndrome. Neurobiol. Dis. 134:104622. doi: 10.1016/j.nbd.2019.104622, PMID: 31698054

[ref41] Kullmann D. M. Lamsa K. P. (2007). Long-term synaptic plasticity in hippocampal interneurons. Nat. Rev. Neurosci. 8, 687–699. doi: 10.1038/nrn2207 17704811

[ref42] Lee F. H. F. Lai T. K. Y. Su P. Liu F. (2019). Altered cortical cytoarchitecture in the Fmr1 knockout mouse. Mol. Brain 12:56. doi: 10.1186/s13041-019-0478-8, PMID: 31200759 PMC6570929

[ref43] Lovelace J. W. Ethell I. M. Binder D. K. Razak K. A. (2018). Translation-relevant EEG phenotypes in a mouse model of fragile X syndrome. Neurobiol. Dis. 115, 39–48. doi: 10.1016/j.nbd.2018.03.012, PMID: 29605426 PMC5969806

[ref44] Lovelace J. W. Rais M. Palacios A. R. Shuai X. S. Bishay S. Popa O.. (2020). Deletion of Fmr1 from forebrain excitatory neurons triggers abnormal cellular, EEG, and behavioral phenotypes in the auditory cortex of a mouse model of fragile X syndrome. Cereb. Cortex 30, 969–988. doi: 10.1093/cercor/bhz141, PMID: 31364704 PMC7132927

[ref45] Luhmann H. J. Fukuda A. Kilb W. (2015). Control of cortical neuronal migration by glutamate and GABA. Front. Cell. Neurosci. 9:4. doi: 10.3389/fncel.2015.00004 25688185 PMC4311642

[ref46] Maurin T. Zongaro S. Bardoni B. (2014). Fragile X syndrome: from molecular pathology to therapy. Neurosci. Biobehav. Rev. 46, 242–255. doi: 10.1016/j.neubiorev.2014.01.006 24462888

[ref47] Morin-Parent F. Champigny C. Lacroix A. Corbin F. Lepage J.-F. (2019). Hyperexcitability and impaired intracortical inhibition in patients with fragile-X syndrome. Transl. Psychiatry 9:312. doi: 10.1038/s41398-019-0650-z, PMID: 31748507 PMC6868148

[ref48] Nolan S. O. Reynolds C. D. Smith G. D. Holley A. J. Escobar B. Chandler M. A.. (2017). Deletion of Fmr1 results in sex-specific changes in behavior. Brain Behav. 7:e00800. doi: 10.1002/brb3.800, PMID: 29075560 PMC5651384

[ref49] Nomura T. Musial T. F. Marshall J. J. Zhu Y. Remmers C. L. Xu J.. (2017). Delayed maturation of fast-spiking interneurons is rectified by activation of the TrkB receptor in the mouse model of fragile X syndrome. J. Neurosci. 37, 11298–11310. doi: 10.1523/JNEUROSCI.2893-16.2017, PMID: 29038238 PMC5700416

[ref50] Olmos-Serrano J. L. Corbin J. G. Burns M. P. (2011). The GABAA receptor agonist THIP ameliorates specific behavioral deficits in the mouse model of fragile X syndrome. Dev. Neurosci. 33, 395–403. doi: 10.1159/000332884, PMID: 22067669 PMC3254038

[ref51] Olmos-Serrano J. L. Paluszkiewicz S. M. Martin B. S. Kaufmann W. E. Corbin J. G. Huntsman M. M. (2010). Defective GABAergic neurotransmission and pharmacological rescue of neuronal hyperexcitability in the amygdala in a mouse model of fragile X syndrome. J. Neurosci. 30, 9929–9938. doi: 10.1523/JNEUROSCI.1714-10.2010, PMID: 20660275 PMC2948869

[ref52] Palumbo J. M. Thomas B. F. Budimirovic D. Siegel S. Tassone F. Hagerman R.. (2023). Role of the endocannabinoid system in fragile X syndrome: potential mechanisms for benefit from cannabidiol treatment. J. Neurodev. Disord. 15:1. doi: 10.1186/s11689-023-09475-z, PMID: 36624400 PMC9830713

[ref53] Paluszkiewicz S. M. Martin B. S. Huntsman M. M. (2011a). Fragile X syndrome: the GABAergic system and circuit dysfunction. Dev. Neurosci. 33, 349–364. doi: 10.1159/000329420, PMID: 21934270 PMC3254035

[ref54] Paluszkiewicz S. M. Olmos-Serrano J. L. Corbin J. G. Huntsman M. M. (2011b). Impaired inhibitory control of cortical synchronization in fragile X syndrome. J. Neurophysiol. 106, 2264–2272. doi: 10.1152/jn.00421.2011, PMID: 21795626 PMC3214096

[ref55] Patel A. B. Hays S. A. Bureau I. Huber K. M. Gibson J. R. (2013). A target cell-specific role for presynaptic Fmr1 in regulating glutamate release onto neocortical fast-spiking inhibitory neurons. J. Neurosci. 33, 2593–2604. doi: 10.1523/jneurosci.2447-12.2013, PMID: 23392687 PMC3711607

[ref56] Pirbhoy P. S. Rais M. Lovelace J. W. Woodard W. Razak K. A. Binder D. K.. (2020). Acute pharmacological inhibition of matrix metalloproteinase-9 activity during development restores perineuronal net formation and normalizes auditory processing in Fmr1 KO mice. J. Neurochem. 155, 538–558. doi: 10.1111/jnc.15037, PMID: 32374912 PMC7644613

[ref57] Pouchelon G. Dwivedi D. Bollmann Y. Agba C. K. Xu Q. Mirow A. M. C.. (2021). The organization and development of cortical interneuron presynaptic circuits are area specific. Cell Rep. 37:109993. doi: 10.1016/j.celrep.2021.109993, PMID: 34758329 PMC8832360

[ref58] Prager E. M. Bergstrom H. C. Wynn G. H. Braga M. F. (2016). The basolateral amygdala γ-aminobutyric acidergic system in health and disease. J. Neurosci. Res. 94, 548–567. doi: 10.1002/jnr.23690, PMID: 26586374 PMC4837071

[ref59] Rais M. Lovelace J. W. Shuai X. S. Woodard W. Bishay S. Estrada L.. (2022). Functional consequences of postnatal interventions in a mouse model of fragile X syndrome. Neurobiol. Dis. 162:105577. doi: 10.1016/j.nbd.2021.105577 34871737

[ref60] Real R. Peter M. Trabalza A. Khan S. Smith M. A. Dopp J.. (2018). In vivo modeling of human neuron dynamics and down syndrome. Science 362:eaau1810. doi: 10.1126/science.aau1810, PMID: 30309905 PMC6570619

[ref61] Reinhard S. M. Rais M. Afroz S. Hanania Y. Pendi K. Espinoza K.. (2019). Reduced perineuronal net expression in Fmr1 KO mice auditory cortex and amygdala is linked to impaired fear-associated memory. Neurobiol. Learn. Mem. 164:107042. doi: 10.1016/j.nlm.2019.107042, PMID: 31326533 PMC7519848

[ref62] Reyes S. T. Mohajeri S. Krasinska K. Guo S. G. Gu M. Pisani L.. (2020). GABA measurement in a neonatal fragile X syndrome mouse model using (1)H-magnetic resonance spectroscopy and mass spectrometry. Front. Mol. Neurosci. 13:612685. doi: 10.3389/fnmol.2020.612685, PMID: 33390902 PMC7775297

[ref63] Richter J. D. Zhao X. (2021). The molecular biology of FMRP: new insights into fragile X syndrome. Nat. Rev. Neurosci. 22, 209–222. doi: 10.1038/s41583-021-00432-0, PMID: 33608673 PMC8094212

[ref64] Rotschafer S. Razak K. (2013). Altered auditory processing in a mouse model of fragile X syndrome. Brain Res. 1506, 12–24. doi: 10.1016/j.brainres.2013.02.038, PMID: 23458504

[ref65] Schaefer T. L. Davenport M. H. Erickson C. A. (2015). Emerging pharmacologic treatment options for fragile X syndrome. Appl. Clin. Genet. 8, 75–93. doi: 10.2147/TACG.S35673 25897255 PMC4396424

[ref66] Sears S. M. Hewett S. J. (2021). Influence of glutamate and GABA transport on brain excitatory/inhibitory balance. Exp. Biol. Med. 246, 1069–1083. doi: 10.1177/1535370221989263, PMID: 33554649 PMC8113735

[ref67] Selby L. Zhang C. Sun Q. Q. (2007). Major defects in neocortical GABAergic inhibitory circuits in mice lacking the fragile X mental retardation protein. Neurosci. Lett. 412, 227–232. doi: 10.1016/j.neulet.2006.11.062, PMID: 17197085 PMC1839948

[ref68] Sidhu H. Dansie L. E. Hickmott P. W. Ethell D. W. Ethell I. M. (2014). Genetic removal of matrix metalloproteinase 9 rescues the symptoms of fragile X syndrome in a mouse model. J. Neurosci. 34, 9867–9879. doi: 10.1523/JNEUROSCI.1162-14.2014, PMID: 25057190 PMC4107404

[ref69] Sohal V. S. Rubenstein J. L. R. (2019). Excitation-inhibition balance as a framework for investigating mechanisms in neuropsychiatric disorders. Mol. Psychiatry 24, 1248–1257. doi: 10.1038/s41380-019-0426-0, PMID: 31089192 PMC6742424

[ref70] Tong L. M. Djukic B. Arnold C. Gillespie A. K. Yoon S. Y. Wang M. M.. (2014). Inhibitory interneuron progenitor transplantation restores normal learning and memory in ApoE4 knock-in mice without or with Aβ accumulation. J. Neurosci. 34, 9506–9515. doi: 10.1523/JNEUROSCI.0693-14.2014, PMID: 25031394 PMC4099537

[ref71] Tuncdemir S. N. Wamsley B. Stam F. J. Osakada F. Goulding M. Callaway E. M.. (2016). Early somatostatin interneuron connectivity mediates the maturation of deep layer cortical circuits. Neuron 89, 521–535. doi: 10.1016/j.neuron.2015.11.020, PMID: 26844832 PMC4861073

[ref72] van der Molen M. J. Stam C. J. van der Molen M. W. (2014). Resting-state EEG oscillatory dynamics in fragile X syndrome: abnormal functional connectivity and brain network organization. PLoS One 9:e88451. doi: 10.1371/journal.pone.0088451, PMID: 24523898 PMC3921158

[ref73] Van der Molen M. J. Van der Molen M. W. (2013). Reduced alpha and exaggerated theta power during the resting-state EEG in fragile X syndrome. Biol. Psychol. 92, 216–219. doi: 10.1016/j.biopsycho.2012.11.013, PMID: 23182872

[ref74] Veenstra-VanderWeele J. Cook E. H. King B. H. Zarevics P. Cherubini M. Walton-Bowen K.. (2017). Arbaclofen in children and adolescents with autism spectrum disorder: a randomized, controlled, phase 2 trial. Neuropsychopharmacology 42, 1390–1398. doi: 10.1038/npp.2016.237 27748740 PMC5436109

[ref75] Vislay R. L. Martin B. S. Olmos-Serrano J. L. Kratovac S. Nelson D. L. Corbin J. G.. (2013). Homeostatic responses fail to correct defective amygdala inhibitory circuit maturation in fragile X syndrome. J. Neurosci. 33, 7548–7558. doi: 10.1523/JNEUROSCI.2764-12.2013, PMID: 23616559 PMC3684185

[ref76] Wang J. Ethridge L. E. Mosconi M. W. White S. P. Binder D. K. Pedapati E. V.. (2017). A resting EEG study of neocortical hyperexcitability and altered functional connectivity in fragile X syndrome. J. Neurodev. Disord. 9:11. doi: 10.1186/s11689-017-9191-z, PMID: 28316753 PMC5351111

[ref77] Wen T. H. Afroz S. Reinhard S. M. Palacios A. R. Tapia K. Binder D. K.. (2018a). Genetic reduction of matrix metalloproteinase-9 promotes formation of perineuronal nets around parvalbumin-expressing interneurons and normalizes auditory cortex responses in developing Fmr1 Knock-out mice. Cereb. Cortex 28, 3951–3964. doi: 10.1093/cercor/bhx258, PMID: 29040407 PMC6188540

[ref78] Wen T. H. Binder D. K. Ethell I. M. Razak K. A. (2018b). The perineuronal 'safety' net? Perineuronal net abnormalities in neurological disorders. Front. Mol. Neurosci. 11:270. doi: 10.3389/fnmol.2018.00270, PMID: 30123106 PMC6085424

[ref79] Wen T. H. Lovelace J. W. Ethell I. M. Binder D. K. Razak K. A. (2019). Developmental changes in EEG phenotypes in a mouse model of fragile X syndrome. Neuroscience 398, 126–143. doi: 10.1016/j.neuroscience.2018.11.047, PMID: 30528856 PMC6331246

[ref80] Willerman L. Schultz R. Rutledge J. N. Bigler E. D. (1991). In vivo brain size and intelligence. Intelligence 15, 223–228. doi: 10.1016/0160-2896(91)90031-8

[ref81] Yang Y. M. Arsenault J. Bah A. Krzeminski M. Fekete A. Chao O. Y.. (2020). Identification of a molecular locus for normalizing dysregulated GABA release from interneurons in the fragile X brain. Mol. Psychiatry 25, 2017–2035. doi: 10.1038/s41380-018-0240-0, PMID: 30224722 PMC7473840

[ref82] Zhang W. Xiong B. R. Zhang L. Q. Huang X. Yuan X. Tian Y. K.. (2021). The role of the GABAergic system in diseases of the central nervous system. Neuroscience 470, 88–99. doi: 10.1016/j.neuroscience.2021.06.037 34242730

[ref83] Zhu B. Eom J. Hunt R. F. (2019). Transplanted interneurons improve memory precision after traumatic brain injury. Nat. Commun. 10:5156. doi: 10.1038/s41467-019-13170-w, PMID: 31727894 PMC6856380

